# Analytical and clinical evaluation of the Alinity m Resp-4-Plex assay in comparison to two singleplex SARS-CoV-2 assays and one multiplex respiratory assay

**DOI:** 10.1128/spectrum.00306-25

**Published:** 2026-03-10

**Authors:** Robert Ehret, Malik Prentice, Birgit Reinhardt, Martin Obermeier

**Affiliations:** 1 Medical Center for Infectious Diseases Berlin, Berlin, Germany; 2 Abbott GmbH 163051 , Wiesbaden, Germany; National Microbiology Laboratory, Winnipeg, Manitoba, Canada

**Keywords:** SARS-CoV-2, influenza A, influenza B, RSV, real-time PCR, Alinity m

## Abstract

After transition to the endemic phase of severe acute respiratory syndrome coronavirus 2 (SARS-CoV-2) infections, multiplex molecular assays detecting SARS-CoV-2, influenza A (FluA) and B (FluB), and respiratory syncytial virus (RSV) are key to following international monitoring guidance, especially in future outbreak situations. In the present study, the analytical and clinical performance of the Alinity m Resp-4-Plex assay (“Alin4Plex”) was evaluated in comparison to those of three other assays: Alinity m SARS-CoV-2 (“AlinSARS”), RealTi*m*e SARS-CoV-2 (“RT-SARS”), and Allplex SARS-CoV-2/FluA/FluB/RSV (“Allplex”). Alin4Plex and AlinSARS exhibited 100% detection rates at 100 IU/mL, while RT-SARS and Allplex showed 90 and 40% at 200 IU/mL, respectively. Similarly, 100% detection rates by Alin4Plex were obtained at 100 (FluA, FluB) and 500 cp/mL (RSV) and by Allplex at 5,000 (FluB) and 10,000 cp/mL (RSV), with a 90% detection rate at 10,000 cp/mL (FluA). Retesting clinical specimens with 201, 198, 73, and 188 positive results for SARS-CoV-2, FluA, FluB, and RSV using Alin4Plex, the positivity rate by Allplex was considerably lower, with 119 (59%), 154 (78%), 42 (58%), and 91 positive specimens (48%), respectively, and decreased with increasing Ct values. Ct values correlated well between Alin4Plex and Allplex for SARS-CoV-2 and RSV (*r* > 0.92), but correlation was only moderate for FluA and FluB (*r* = 0.56 and 0.40, respectively). Overall, the multiplex assay Alin4Plex conducted on the high-throughput, random access Alinity m system exhibited high sensitivity for SARS-CoV-2, FluA, FluB, and RSV in a single test. It enables laboratories to rapidly scale up respiratory testing in future outbreak situations and provides valuable information for optimized patient management.

**IMPORTANCE:**

After the transition of the coronavirus disease 2019 (COVID-19) pandemic into an endemic phase, a rebound of transmissions of influenza A, influenza B, and respiratory syncytial virus (RSV) with the risk of severe morbidity and mortality has been observed. Overlapping symptoms complicate accurate diagnosis of respiratory infections and may delay adequate treatment. A multiplex PCR assay conducted on a random-access high-throughput automated platform enables accelerated and simultaneous detection of respiratory viruses, thus enhancing diagnostic efficiency, reducing the need for multiple tests, consolidating workflow, and enabling timely and appropriate patient care. This study evaluated the analytical and clinical performance of the Alinity m Resp-4-Plex assay for detection of severe acute respiratory syndrome coronavirus 2 (SARS-CoV-2), influenza A, influenza B, and RSV in comparison to other commercially available multiplex and singleplex tests. Results are crucial for identifying the most appropriate diagnostic method to improve patient outcome and optimize resource utilization in clinical settings.

## INTRODUCTION

In 2020, the newly emerged severe acute respiratory syndrome coronavirus 2 (SARS-CoV-2) spread across the world and caused significant morbidity and mortality. While non-pharmaceutical interventions, such as stay-at-home orders, social distancing, and personal protective measures, were effective against SARS-CoV-2, these measures also disrupted the transmission of common respiratory virus infections like influenza A (FluA), influenza B (FluB), and respiratory syncytial virus (RSV) (1). Now, after SARS-CoV-2 has become endemic, and non-pharmaceutical interventions were ended, a rebound of influenza and RSV is observed that may even exceed normal levels (2–4). Symptoms of RSV infections range from a mild cold to severe symptoms of influenza. Especially in infants, elderly, or immunocompromised people, RSV infections account for severe illness, including death (1, 5). Also, seasonal influenza leads to significant annual morbidity and mortality (1, 6). Therefore, in a joint statement, European Commission, World Health Organization (WHO), and European Center for Disease Prevention and Control (ECDC) recommended monitoring not only the spread of SARS-CoV-2 but also the transmission of FluA, FluB, and RSV (7).

Due to indistinguishable symptoms, it is not possible to reliably differentiate between these respiratory viruses only based on the clinical condition of a patient, especially during the early course of disease. Fast and sensitive PCR-based diagnostic tests are, therefore, of major clinical utility for reliable diagnosis. Instead of a stepwise approach, a multiplex PCR assay for the simultaneous detection of SARS-CoV-2, FluA, FluB, and RSV conducted on a high-throughput automated platform allows scalability, saves time and cost, accelerates linkage to clinical intervention and potentially life-saving treatments, and enables integrated surveillance of the four most relevant viruses (8–10).

The present study aimed to evaluate and compare the analytical and clinical performance of the Alinity m Resp-4-Plex assay (“Alin4Plex”) to that of the commercially available singleplex assays Alinity m SARS-CoV-2 (“AlinSARS”) and RealTi*m*e SARS-CoV-2 (“RT-SARS”) and to the multiplex assay Allplex SARS-CoV-2/FluA/FluB/RSV (“Allplex”) frequently used in routine molecular diagnostics. Detection rates for SARS-CoV-2, FluA, FluB, and RSV across the respective assays in standard solutions, as well as the qualitative and quantitative concordance between Alin4Plex and Allplex in a large number of clinical SARS-CoV-2, FluA, FluB, and RSV specimens, were assessed.

## MATERIALS AND METHODS

### Molecular test methods

The Alinity m Resp-4-Plex assay (Abbott Molecular, Inc., Des Plaines, IL, USA) is a multiplex real-time reverse transcription polymerase chain reaction (RT-PCR) test for the qualitative detection and differentiation of RNA from SARS-CoV-2, FluA, FluB, and RSV in nasopharyngeal swabs from individuals with symptoms of respiratory tract infection. Target sequences of the assay are the highly conserved, SARS-CoV-2-specific RdRp and N genes of the SARS-CoV-2 genome, the matrix protein genes in the genomes of RSV and FluA, and the nonstructural 1 gene in the FluB genome (11). The Alinity m SARS-CoV-2 (12) and the RealTi*m*e SARS-CoV-2 (13) assays (both Abbott Molecular, Inc., Des Plaines, IL, USA) are real-time RT-PCR tests for the qualitative detection of RNA from SARS-CoV-2 in nasopharyngeal and oropharyngeal swabs from patients suspected of coronavirus disease 2019 (COVID-19) infection. Primers and probes of both assays target highly conserved, SARS-CoV-2-specific sequences in the RdRp and N genes of the SARS-CoV-2 genome. Alin4Plex and AlinSARS assays were conducted on the fully automated, high-throughput, random and continuous access Alinity m system (Abbott Molecular, Inc., Des Plaines, IL, USA), which allows priority sample processing. RT-SARS was performed in a batchwise processing on the *m*2000 system (Abbott Molecular, Inc., Des Plaines, IL, USA) using the *m*2000sp for sample extraction and the *m*2000rt for PCR amplification and detection. Allplex SARS-CoV-2/FluA/FluB/RSV (Seegene, Inc., Seoul, South Korea) is a multiplex real-time RT-PCR assay for detecting SARS-CoV-2, FluA, FluB, and RSV in nasopharyngeal and oropharyngeal swabs and sputum (14).

Batchwise processing consisted of extraction and master-mix preparation on the Seegene NIMBUS automated liquid handling workstation. Amplification and detection were conducted on the CFX96 Touch Real-Time PCR Detection System (Bio-Rad Laboratories GmbH, Feldkirchen, Germany). The assay targets the S, RdRp, and N genes of the SARS-CoV-2 genome (14), while the target regions for FluA, FluB, and RSV were not disclosed by the manufacturer.

Details regarding primers and probes of all four assays are proprietary to the manufacturers and not publicly available. Manufacturer’s instructions were followed for all assays.

### Detection rates for SARS-CoV-2

To compare the detection rates of Alin4Plex for SARS-CoV-2 with that of the other three assays, a dilution series of the 1st WHO International Standard for SARS-CoV-2 RNA (NIBSC code: 20/146; 7.70 log IU/mL [15, 16]) was prepared. The material was first reconstituted in 0.5 mL molecular-grade water according to the instructions by the manufacturer and subsequently diluted using saline solution to obtain target concentrations of 200, 100, 50, 25, 10, 5, and 1 IU/mL. Twenty replicates for 25 and 50 IU/mL and 10 replicates for the other concentrations were tested head-to-head by the four assays, and detection rates were assessed. This evaluation focused on comparing detection rates, not determining exact limits of detection (LODs). Thus, the same dilutions within the expected differentiation range were used across all assays.

### Detection rates for FluA, FluB, and RSV

The detection rates for FluA, FluB, and RSV were assessed using quantitative genomic RNA from FluA virus (H1N1) strain A/PR/8/34 (ATCC VR-95DQ; "ATCC-FluA"), FluB virus (Victoria Lineage) strain B/Florida/78/2015 (ATCC VR-1931DQ; "ATCC-FluB"), and human RSV strain A2 (ATCC VR-1540DQ; "ATCC-RSV") (American Type Culture Collection [ATCC], Manassas, VA, USA). The standard solutions were serially diluted with saline to concentrations of 10,000, 5,000, 1,000, 500, 200, 100, 50, 25, 10, and 5 copies/mL (cp/mL), as applicable. Ten replicates of each concentration were tested by Alin4Plex and compared head-to-head with Allplex. The evaluation aimed to investigate differences in sensitivity rather than determining exact LODs. Therefore, identical dilutions in the expected differentiation range were used for both assays.

### Clinical performance of Alin4Plex and Allplex

Nine hundred and fifty-four nasopharyngeal routine specimens with sufficient residual volume, including 654 samples positive for either SARS-CoV-2, FluA, FluB, or RSV, and 300 negative samples were selected based on their Alin4Plex result during routine diagnostics. SARS-CoV-2-positive samples were collected between October 2021 and January 2022 and could be retested without freeze/thaw cycles. Due to the low circulation of flu and RSV during the winter season of 2020/2021, the collection period was extended for FluA (December 2021–December 2022), FluB (January 2022–April 2023; two archived samples from January 2020 were also included), and RSV (September 2021–December 2022; plus one archived sample from January 2020). Samples were stored frozen at −20°C for up to 15 months (three samples for 39 months) before being thawed and batchwise retested with Allplex. Qualitative results and Ct values for the four respiratory viruses were compared between Alin4Plex and Allplex. Only residual samples from routine respiratory virus testing were used.

### Statistical analysis

To compare SARS-CoV-2 results between Allplex and Alin4Plex, the Allplex Ct values for the three SARS-CoV-2 target regions were combined in a mean Ct value. The correlation between Alin4Plex and Allplex was assessed by Deming regression, Pearson’s correlation coefficient *r*, and Bland-Altman analysis. Detection rates were evaluated using probit regression analysis with a maximum likelihood estimation according to the Clinical and Laboratory Standards Institute (CLSI) guideline EP17-A2 (17). Detection rates of 95% were estimated by extrapolating the probit regression graph to 6.645 probit. All statistical evaluations were conducted with Microsoft Excel and the add-in software, Analyse-it.

## RESULTS

### Detection rates for SARS-CoV-2

A dilution series of the 1st WHO International Standard for SARS-CoV-2 RNA with six target concentrations ranging between 200 and 5 IU/mL was tested in replicates of 10 or 20 using Alin4Plex, AlinSARS, RT-SARS, and Allplex, respectively. Additionally, a dilution with a target concentration of 1 IU/mL was tested with Alin4Plex. The highest detection rates were found with Alin4Plex and AlinSARS (both 100% at 200 and 100 IU/mL), followed by RT-SARS (90% at 200 IU/mL) and Allplex (40% at 200 IU/mL) (Table 1). Probit regression analyses were performed and yielded estimated LODs (95% detection rates) for Alin4Plex of 56 IU/mL (1.75 log IU/mL), for AlinSARS of 88 IU/mL (1.94 log IU/mL), for RT-SARS of 238 IU/mL (2.38 log IU/mL), and for Allplex of 407 IU/mL (2.61 log IU/mL), suggesting that Alin4Plex has the highest sensitivity for SARS-CoV-2 (Table 1; Fig. S1)*.*

**TABLE 1:** Detection rates by Alin4Plex, AlinSARS, RT-SARS, and Allplex using a dilution series of the 1st WHO International Standard for SARS-CoV-2 RNA and estimated LODs after probit regression analysis^*a*^

Target concentration (IU/mL)	*N*	Alin4Plex		AlinSARS		RT-SARS		Allplex	
		Number of positive replicates	Detection rate	Number of positive replicates	Detection rate	Number of positive replicates	Detection rate	Number of positive replicates	Detection rate
200	10	10	100%	10	100%	9	90%	4	40%
100	10	10	100%	10	100%	5	50%	2	20%
50	20	17	89%^*b*^	12	60%	7	35%	1	5%
25	20	9	45%	5	25%	5	25%	0	0%
10	10	1	10%	0	0%	2	20%	0	0%
5	10	1	10%	0	0%	0	0%	0	0%
1	10	0	0%	–	–	–	–	–	–
Estimated LOD		56 IU/mL (1.75 log IU/mL)		88 IU/mL (1.94 log IU/mL)		238 IU/mL (2.38 log IU/mL)		407 IU/mL (2.61 log IU/mL)	

^*a*^ *N*, number of replicates tested; –, not tested.

^*b*^ One replicate had an invalid result.

### Detection rates for FluA, FluB, and RSV

Serial dilutions of ATCC standards for FluA, FluB, and RSV were tested with Alin4Plex and Allplex. Alin4Plex exhibited consistent detection rates of 100% down to 100 (FluA and FluB) and 500 cp/mL (RSV) (Table 2). In contrast, detection rates with Allplex were 90% at 10,000 cp/mL (FluA) and 100% at 5,000 (FluB) and 10,000 cp/mL (RSV) (Table 3). Estimated 95% detection rates by probit regression analyses for Alin4Plex were at 109 (2.04 log cp/mL), 59 (1.77 log cp/mL), and 309 cp/mL (2.49 log cp/mL) for FluA, FluB, and RSV, respectively, while the corresponding results for Allplex were 13,538 (4.13 log cp/mL), 1,779 (3.25 log cp/mL), and 5,700 cp/mL (3.76 log cp/mL) (Tables 2 and 3; Fig. S2)*.*

**TABLE 2:** Detection rates by Alin4Plex using dilution series of ATCC-FluA, ATCC-FluB, and ATCC-RSV and estimated LODs after probit regression analysis^*a*^

Target concentration (cp/mL)	*N*	FluA		FluB		RSV	
		Number of positive replicates	Detection rate	Number of positive replicates	Detection rate	Number of positive replicates	Detection rate
10,000	10	10	100%	10	100%	10	100%
5,000	10	10	100%	10	100%	10	100%
1,000	10	10	100%	10	100%	10	100%
500	10	10	100%	10	100%	10	100%
200	10	10	100%	10	100%	8	80%
100	10	10	100%	10	100%	6	60%
50	10	8	80%	9	90%	4	40%
25	10	8	80%	10	100%	–	–
10	10	9	90%	6	60%	–	–
5	10	4	40%	3	30%	–	–
Estimated LOD (Alin4Plex)		109 cp/mL (2.04 log cp/mL)		59 cp/mL (1.77 log cp/mL)		309 cp/mL (2.49 log cp/mL)	

^*a*^ *N*, number of replicates tested; –, not tested.

**TABLE 3:** Detection rates by Allplex using dilution series of ATCC-FluA, ATCC-FluB, and ATCC-RSV and estimated LODs after probit regression analysis^*a*^

Target concentration (cp/mL)	*N*	FluA		FluB		RSV	
		Number of positive replicates	Detection rate	Number of positive replicates	Detection rate	Number of positive replicates	Detection rate
10,000	10	9	90%	10	100%	10	100%
5,000	10	6	60%	10	100%	9	90%
1,000	10	3	30%	6	60%	2	20%
500	10	1	10%	4	40%	2	20%
200	10	0	0%	1	10%	0	0%
100	10	0	0%	0	0%	0	0%
50	10	0	0%	1	10%	0	0%
25	10	0^*b*^	0%	1	10%	–	–
10	10	0	0%	0	0%	–	–
5	10	0	0%	0	0%	–	–
Estimated LOD (Allplex)		13,538 cp/mL (4.13 log cp/mL)		1,779 cp/mL (3.25 log cp/mL)		5,700 cp/mL (3.76 log cp/mL)	

^*a*^ *N*, number of replicates tested; –, not tested.

^*b*^ One replicate had an invalid result.

### Clinical performance of Alin4Plex and Allplex

Overall, 954 nasopharyngeal routine specimens tested with Alin4Plex were retested with Allplex. Three hundred specimens negative for SARS-CoV-2, FluA, FluB, and RSV with Alin4Plex were also negative during retesting with Allplex. The other 654 specimens provided 201, 198, 73, and 188 positive results for SARS-CoV-2, FluA, FluB, and RSV by Alin4Plex, respectively, including six samples with co-infections: five specimens were positive for FluA and RSV, and one specimen was positive for SARS-CoV-2 and RSV. All positive results were categorized according to their Alin4Plex Ct values: below 25 Ct, between 25 and 30 Ct, between 30 and 35 Ct, and above 35 Ct. For each category, the detection rates by Allplex in comparison to Alin4Plex were determined. The results are shown in Table 4. In the category with Ct values below 25 with Alin4Plex, the positivity rates by Allplex were high with 95–100% for all four pathogens. In the subsequent category with Ct values 25–30 by Alin4Plex, the positivity rates by Allplex were still high with 94 and 100% for SARS-CoV-2 and RSV, respectively, but considerably decreased to 84% for FluA and only 26% for FluB. In the third category (Ct values 30–35 with Alin4Plex), the positivity rates with Allplex ranged between 22 and 68%, while they were low (0–12%) in the last category with Ct values above 35 by Alin4Plex. Overall, Allplex detected only 59, 78, 58, and 48% of all positive SARS-CoV-2, FluA, FluB, and RSV specimens, respectively. Notably, 10 SARS-CoV-2, two FluA, and one RSV sample of the above specimens could be retested not only with Allplex but also with Alin4Plex in parallel due to sufficient residual volume. Results confirmed the differences in detection rates (Table S1). In addition, five of the six co-infected samples were concordantly detected by both assays, while one specimen positive for FluA and RSV with Alin4Plex (Ct values 36.6 and 34.9, respectively) was negative for both viruses with Allplex.

**Fig 1:**
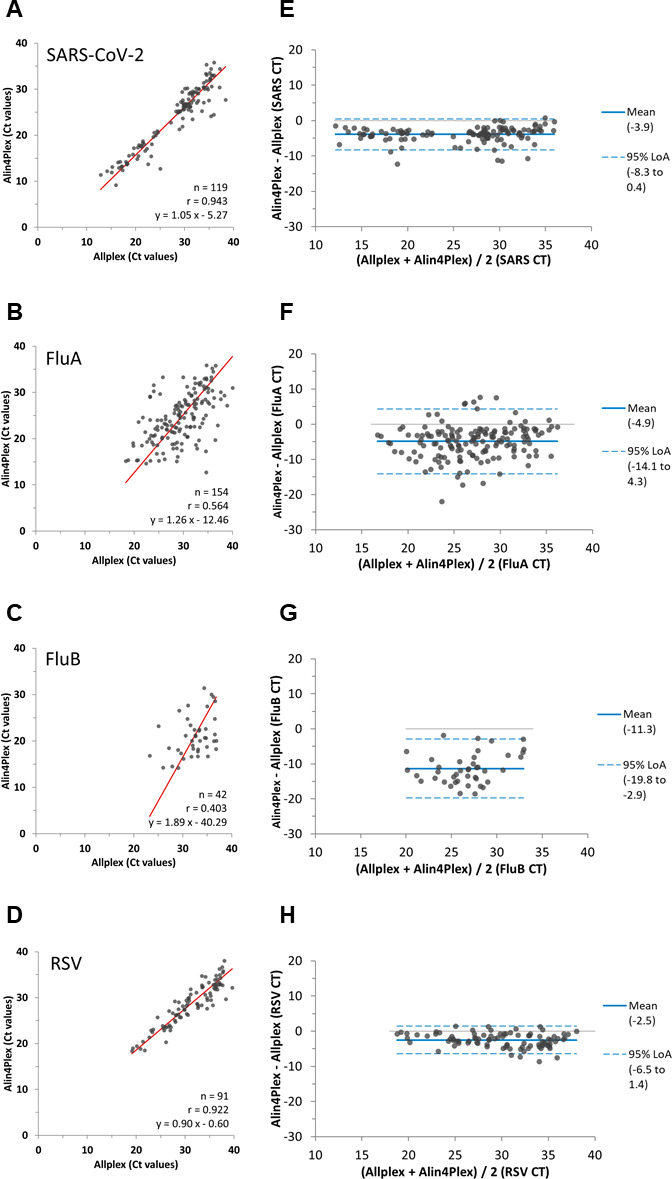
Ct value correlation between Alin4Plex and Allplex in nasopharyngeal routine specimens. Deming regression and Pearson’s correlation coefficients *r* are shown for (**A**) SARS-CoV-2 (*n* = 119), (**B**) FluA (*n* = 154), (**C**) FluB (*n* = 42), and (**D**) RSV (*n* = 91). Results of Bland-Altman analyses are shown for (**E**) SARS-CoV-2, (**F**) FluA, (**G**) FluB, and (**H**) RSV.

The correlations between the Ct values by Alin4Plex and Allplex for the four pathogens were evaluated for samples positive by both assays, including 119 SARS-CoV-2, 154 FluA, 42 FluB, and 91 RSV results. Deming regression exhibited a high correlation between Alin4Plex and Allplex for SARS-CoV-2 and RSV with Pearson’s correlation coefficients *r* of 0.943 and 0.922 (Fig. 1A and D) and mean biases of −3.9 and −2.5 Ct values (Alin4Plex – Allplex), respectively (Fig. 1E and H). In contrast, the Ct values for FluA and FluB were less correlated with Pearson’s correlation coefficients *r* of 0.564 and 0.403, respectively (Fig. 1B and C). The corresponding mean biases were −4.9 and −11.3 Ct values (Fig. 1F and G).

**TABLE 4:** Positivity rates of Allplex in clinical nasopharyngeal routine samples that tested positive with Alin4Plex^*a*^^,*b*^

Category of Ct values (Alin4Plex)	SARS-CoV-2			FluA			FluB			RSV		
	*N*	Positive by Allplex		*N*	Positive by Allplex		*N*	Positive by Allplex		*N*	Positive by Allplex	
		*n*	Rate		*n*	Rate		*n*	Rate		*n*	Rate
<25	47	47	100%	84	83	99%	37	35	95%	21	21	100%
25–30	49	46	94%	49	43	84%	19	5	26%	33	33	100%
30–35	54	24	44%	39	25	68%	9	2	22%	56	32	57%
>35	51	2	4%	26	3	12%	8	0	0%	78	5	6%
Total	201	119	59%	198	154	78%	73	42	58%	188	91	48%

^*a*^ *N*, number of positive results by Alin4Plex; *n*, number of positive results by Allplex.

^*b*^ Results were categorized according to the Ct values of Alin4Plex.

## DISCUSSION

International guidance by WHO and ECDC recommends integrated surveillance of SARS-CoV-2, FluA, FluB, and RSV (7). Multiplex PCR assays detecting all four viruses in a single test enable scalability to react in an outbreak situation. In the present study, we evaluated the analytical and clinical performance of the respiratory multiplex assay Alin4Plex in comparison to that of another multiplex assay. In addition, we compared its SARS-CoV-2 performance to that of two singleplex SARS-CoV-2 assays.

In the analytical part of this study, multiplex Alin4Plex exhibited slightly higher SARS-CoV-2 detection rates than singleplex AlinSARS and RT-SARS and considerably higher detection rates than multiplex Allplex using a dilution series of the 1st WHO International Standard for SARS-CoV-2 RNA. This finding is in line with those of previous studies supporting a high sensitivity of Alin4Plex, AlinSARS, and RT-SARS by evaluating their LODs or comparing their sensitivity to multiple other commercially available assays (8, 18–26). Concordantly, a previous study exhibited 100% detection rates for AlinSARS and RT-SARS at 50 cp/mL, while at lower concentrations, AlinSARS showed slightly higher detection rates than RT-SARS (18). Similarly, a recent study found very low LODs with Alin4Plex (22–36 cp/mL for SARS-CoV-2, FluA, FluB, and RSV) compared to the results by two comparators with 32–326 and 58–122 cp/mL, respectively (8).

Evaluating ATCC standard solutions for FluA, FluB, and RSV exhibited a higher sensitivity of Alin4Plex also for these three viruses, showing concentration differences of 1.27–2.09 log cp/mL at the estimated 95% detection rates.

In a previous study, LODs for Allplex were assessed using AccuPlex SARS-CoV-2, Flu A/B, and RSV reference material kits (SeraCare, Milford, MA, USA). Observed LODs were 284–1,650 cp/mL (2.45–3.22 log cp/mL) across the three SARS-CoV-2 targets and 4,917 (3.69 log cp/mL), 249 (2.40 log cp/mL), and 282 cp/mL (2.45 log cp/mL) for FluA, FluB, and RSV, respectively. Like in our study, sensitivity of Allplex for FluA was considerably lower than for FluB and RSV (27). Due to the lack of absolute concentration values in IU/mL, these observed LODs could not directly be compared to our results.

The present study also assessed the clinical performance of Alin4Plex and Allplex using clinical samples positive for SARS-CoV-2, FluA, FluB, or RSV. Alin4Plex showed considerably higher sensitivity for SARS-CoV-2 than Allplex when testing 201 clinical nasopharyngeal routine specimens. Only 59% of the SARS-CoV-2 samples positive by Alin4Plex were also detected positive by Allplex. Positivity rates by Allplex decreased with increasing Alin4Plex Ct values from 100% (Ct values < 25) to 4% (Ct values > 35).

Alin4Plex also exhibited higher positivity rates than Allplex for the other three respiratory viruses. In samples positive for FluA, FluB, or RSV by Alin4Plex, overall positivity rates by Allplex were 78, 58, and 48%, respectively, with decreasing detection rates for increasing Alin4Plex Ct value categories. These findings corresponded well to the differences in detection rates when testing dilutions of the 1st WHO International Standard for SARS-CoV-2 RNA and ATCC standards for FluA, FluB, and RSV with these two assays. Additionally, comparable results were obtained in a similar head-to-head comparison of Alin4Plex, Panther Fusion SARS-CoV-2/FluA/B/RSV (Hologic, Inc., Marlborough, MA, USA), and Allplex RV Master (Seegene, Inc., Seoul, South Korea). Testing of 102, 99, 43, and 93 samples positive for SARS-CoV-2, FluA, FluB, and RSV, respectively, with pre-test Ct values between 15 and 35 yielded a positive result for all specimens with Alin4Plex. In contrast, the Panther Fusion SARS-CoV-2/FluA/B/RSV assay showed overall detection rates for SARS-CoV-2, FluA, FluB, and RSV of 90, 75, 62, and 82%, respectively, while the lowest detection rates of 63, 35, 8, and 49% were observed with Allplex RV Master (28). In a study comparing Allplex on the STARlet-AIOS system (Seegene, Inc., Seoul, South Korea) to Xpert Xpress SARS-CoV-2 plus (“Xpert”; Cepheid, Inc., Sunnyvale, CA, USA), all SARS-CoV-2-positive samples with Ct values ≤35 by Xpert were also detected by Allplex, while only 25% of specimens with Ct values >35 by Xpert were detected by Allplex. This corresponded well to a >1 log higher LOD for Allplex (640 cp/mL, 2.81 log cp/mL) compared to Xpert (32 cp/mL, 1.51 log cp/mL) determined using a serial dilution of an external quality assessment specimen (29). A similar LOD for SARS-CoV-2 on Allplex (650 cp/mL) had also previously been reported (30). Retrospective testing of FluA, FluB, and RSV samples positive for Xpert Xpress SARS-CoV-2/Flu/RSV assay (Cepheid, Inc., Sunnyvale, CA, USA) using Allplex on the STARlet-AIOS system exhibited agreements of 98.3 and 96.7% for FluB and RSV, respectively, but only of 90.1% for FluA. Ct values indicating the concentration levels, however, were not provided (29).

Finally, in a study retesting Allplex-positive samples (20 SARS-CoV-2, 11 FluA, five FluB, and three RSV) with Alin4Plex, all, except one FluB sample with a high Ct value (40.9), were found positive by Alin4Plex. Beyond that, Alin4Plex identified a FluA co-infection (Ct 38.2) not detected by Allplex (18). In another study comparing Alin4Plex with one or multiple comparator assays in the overall 42 patient samples, Alin4Plex not only concordantly identified SARS-CoV-2, FluA, FluB, and RSV but additionally identified one FluA, one FluB, and three RSV infections. All additional findings were confirmed by a second Alinity m and partially confirmed by Cepheid Xpert Xpress SARS-CoV-2/Flu/RSV, suggesting an enhanced sensitivity by Alin4Plex and demonstrating the robust high amplification efficiency, even in the context of a multiplex assay (31).

Alin4Plex Ct values correlated well with Allplex for SARS-CoV-2 and RSV, while the correlation was only moderate for the Ct values of FluA and FluB. Their variability could be caused by differences in extraction efficiency, PCR assay designs, amplicon size, and target regions between the assays. The higher imprecision for FluA and FluB compared to SARS-CoV-2 and RSV does not appear to be related to Alin4Plex since a reproducibility evaluation had exhibited very similar low standard deviations of 0.1–0.5 Ct values across all four pathogens (32).

Overall, studies evaluating the Ct values for all four pathogens across different assays are rare. Good correlation was observed when comparing Ct values by Alin4Plex and Panther Fusion SARS-CoV-2/FluA/B/RSV across all four pathogens (*r* = 0.70 to 0.95); however, like in the present study, the correlation between Ct values by Allplex RV Master and Panther Fusion SARS-CoV-2/FluA/B/RSV was considerably lower for FluA (*r* = 0.28) and FluB (*r* = 0.46) compared to SARS-CoV-2 (*r* = 0.92) and RSV (*r* = 0.76) (28). Two recent studies observed a high correlation of the SARS-CoV-2 Ct values between Alin4Plex and AlinSARS (*R*² ≥ 0.96) (31, 33). In another study comparing Alin4Plex with Xpert Xpress Flu/RSV and Xpert Xpress SARS-CoV-2 (both Cepheid Inc., Sunnyvale, CA, USA), Ct values for all four pathogens correlated well with R² ranging between 0.857 and 0.947 (32). Thus, the above studies suggest that the lower Ct correlations for FluA and FluB in the present study are more likely due to a higher Ct variability of the Allplex assay. Nevertheless, the clinical utility of Ct values remains a matter of debate as method and quality of sample collection may have a major impact on the amount of viral RNA detected (34, 35). Additionally, Ct thresholds for different assays are set independently, e.g. by using unread cycles (19) or incorporating mathematical calculations. Thus, Ct comparisons like the present study may help to avoid potentially misleading interpretation of Ct values and biases. While despite their known limitations, Ct values of SARS-CoV-2 are still used as criteria for active infection and transmissibility in patient management (35), for FluA, FluB, and RSV assays, only qualitative results are reported, whereas their Ct values are of minor importance for patient management and usually not requested.

In the present study, 300 samples were concordantly negative for SARS-CoV-2, FluA, FluB, and RSV with both Alin4Plex and Allplex. Similar results have also been observed with Alin4Plex and two comparator assays showing 100% negative percent agreement (NPA) for all four analytes when testing 20 and 100 negative samples, respectively (8, 28).

A limitation of the study was the low influenza and RSV circulation due to the ongoing SARS-CoV-2 pandemic during the sample collection period in the winter season of 2020/2021. Therefore, the collection period for FluA, FluB, and RSV samples was extended until Apr 2023, and overall three archived samples from the pre-pandemic winter season of 2019/2020 were included in the evaluation. Due to the limited availability of flu and RSV samples, an even distribution across the Ct value categories could not be achieved for these viruses. This may have affected the numerical values of the overall detection rates but did not impact the detection rates per Ct value category.

In case of discordant results, insufficient sample volumes did not allow repeat testing. Thus, an impact of storage until retesting, together with the freeze-thaw cycle, as well as potentially false positive results with Alin4Plex, cannot completely be excluded, especially for samples with high Ct values. However, the impact appears to be limited, as parallel testing of a few samples with Alin4Plex and Allplex had shown similar differences between the two assays. Furthermore, the lower sensitivity of Allplex for the four viruses in clinical specimens was confirmed by parallel testing of dilution series of the 1st WHO International Standard for SARS-CoV-2 RNA, as well as of ATCC standard solutions for FluA, FluB, and RSV. As a limitation, ATCC FluA and RSV nucleic acid molecular standard solutions with defined and sufficiently high concentrations only contained strains that were at least 60 years old. This may have led to a reduced sensitivity of Allplex in comparison to Alin4Plex, though even old strains may re-appear and continue to circulate (36). Thus, modern on-market assays should be able to reliably detect them. Moreover, the limited number of replicates did not allow for a precise estimation of the assays' LODs for the four pathogens, which was beyond the scope of this study in any case. Instead, by using the same concentration levels, probit regression analyses were performed to provide additional evidence regarding the differences in sensitivities across the assays observed when testing clinical specimens.

Another limitation was the evaluation of the Allplex results for SARS-CoV-2 as average Ct values across all three target regions, though in 13 and 30% of the positive samples, Ct values were reported for only one and two targets, respectively. However, the difference between mean Ct values across two or three targets was only 0.74 Ct values (data not shown) and was, therefore, considered negligible.

The analytical and clinical performance of the multiplex Alinity m Resp-4-Plex assay exhibited a higher sensitivity for SARS-CoV-2, FluA, FluB, and RSV than Allplex and even than singleplex SARS-CoV-2 assays when evaluating the 1st WHO International Standard for SARS-CoV-2 RNA, ATCC standard solutions, and a comprehensive number of clinical samples. After the transition to the endemic phase of SARS-CoV-2 infections, testing the four most relevant respiratory viruses in a single, highly sensitive, and fast test on a random access platform can enable laboratories to effectively consolidate their workflow in preparation for future outbreaks and provide important information to clinicians to support optimized patient management.

## SUPPLEMENTAL MATERIAL

Supplemental material spectrum.00306-25-s0001.docx Table S1; Fig. S1 and S2.

ASM does not own the copyrights to Supplemental Material that may be linked to, or accessed through, an article. The authors have granted ASM a non-exclusive, world-wide license to publish the Supplemental Material files. Please contact the corresponding author directly for reuse.
