# Supplementary material for: Analytical and clinical evaluation of the Alinity m Resp-4-Plex assay in comparison to two singleplex SARS-CoV-2 assays and one multiplex respiratory assay
Source: Microbiol Spectr. 2026 Mar 10;14(4):e00306-25. doi: 10.1128/spectrum.00306-25 (PMC13055274; doi:10.1128/spectrum.00306-25)
Supplement: Supplemental material — Table S1; Fig. S1 and S2. [file spectrum.00306-25-s0001.docx]

**Supplemental Material**

| **Category of Ct values (Alin4Plex)** | **SARS-CoV-2** | | |  | **FluA** | | |  | **RSV** | | |
| --- | --- | --- | --- | --- | --- | --- | --- | --- | --- | --- | --- |
|  | **N** | **positive by Allplex** | |  | **N** | **positive by Allplex** | |  | **N** | **positive by Allplex** | |
|  |  | **n** | **Rate** |  |  | **n** | **Rate** |  |  | **n** | **Rate** |
| < 25 | 3 | 3 | 100% |  | 0 | 0 | 0% |  | 0 | 0 | 0% |
| 25 - 30 | 2 | 2 | 100% |  | 0 | 0 | 0% |  | 0 | 0 | 0% |
| 30 - 35 | 3 | 2 | 67% |  | 2 | 0 | 0% |  | 1 | 1 | 100% |
| > 35 | 2 | 0 | 0% |  | 0 | 0 | 0% |  | 0 | 0 | 0% |

**Supplemental Table S1:** Positivity rates of Allplex in selected clinical nasopharyngeal routine samples tested positive with Alin4Plex. These samples had sufficient residual volume to be tested in parallel with both assays. Results were categorized according to the Ct values of Alin4Plex.

N: number of positive results by Alin4Plex; n: number of positive results by Allplex

**Supplemental Figure S1:** Probit regression analyses of the detection rates of A) Alin4Plex, B) AlinSARS, C) RT-SARS, and D) Allplex obtained by using a dilution series of the 1^st^ WHO International Standard for SARS-CoV-2 RNA. Filled circles represent the measured detection rates while open circles indicate estimated 95% detection rates.

**A B**

**
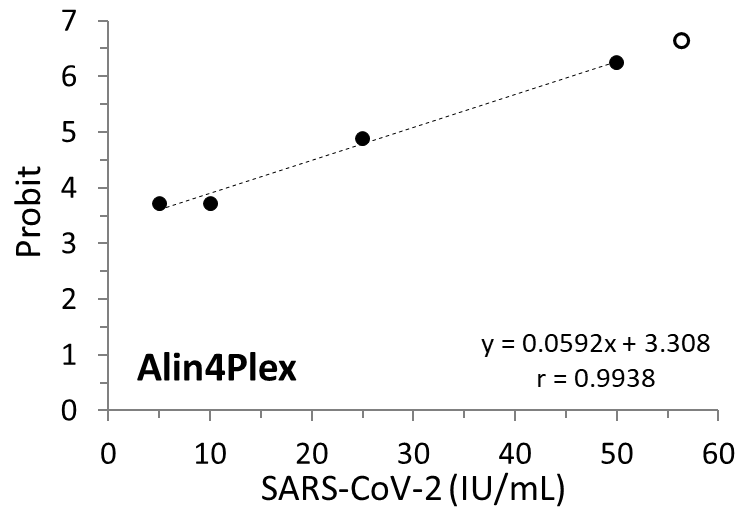

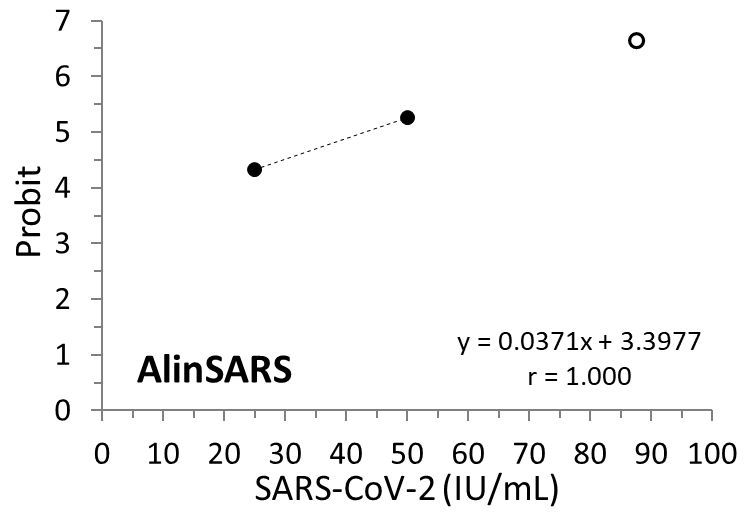
**

**C D**

**
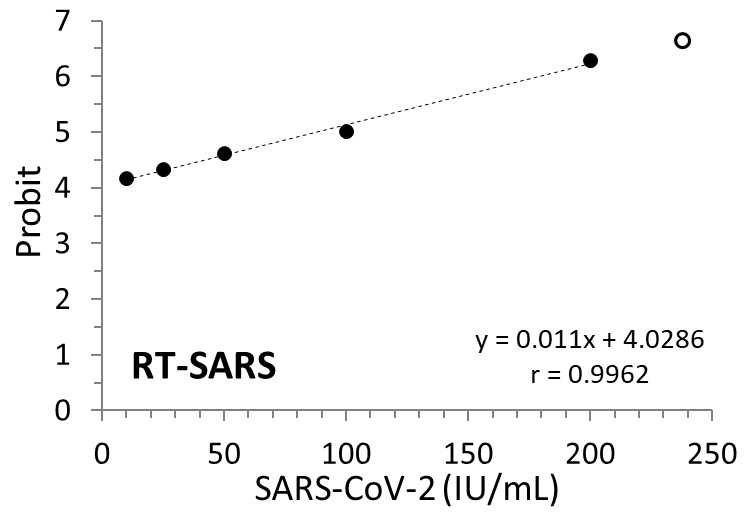

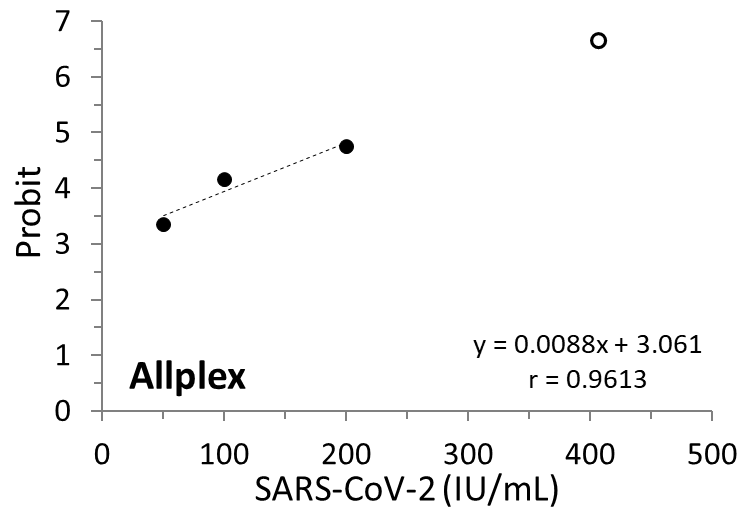
**

**Supplemental Figure S2:** Probit analyses of the detection rates of Alin4Plex for
A) FluA, C) FluB, and E) RSV as well as of Allplex for B) FluA, D) FluB, and F) RSV obtained by using dilution series of ATCC-FluA, ATCC-FluB, and ATCC-RSV, respectively. Filled circles represent the measured detection rates while open circles indicate estimated 95% detection rates.

**A B**

**
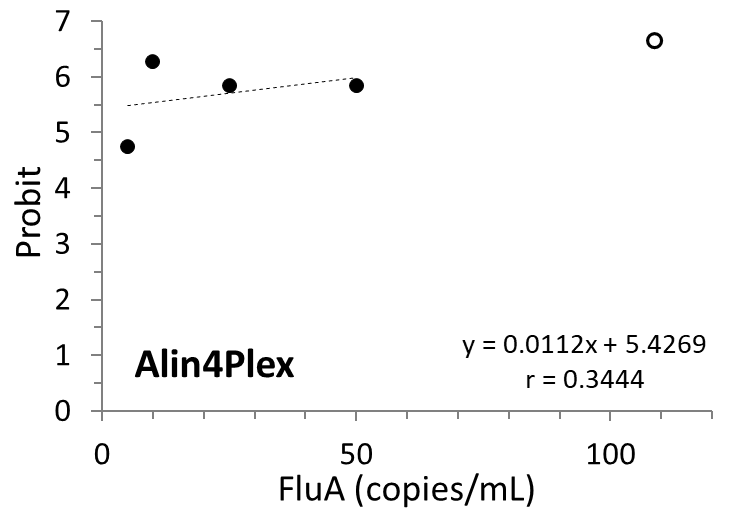

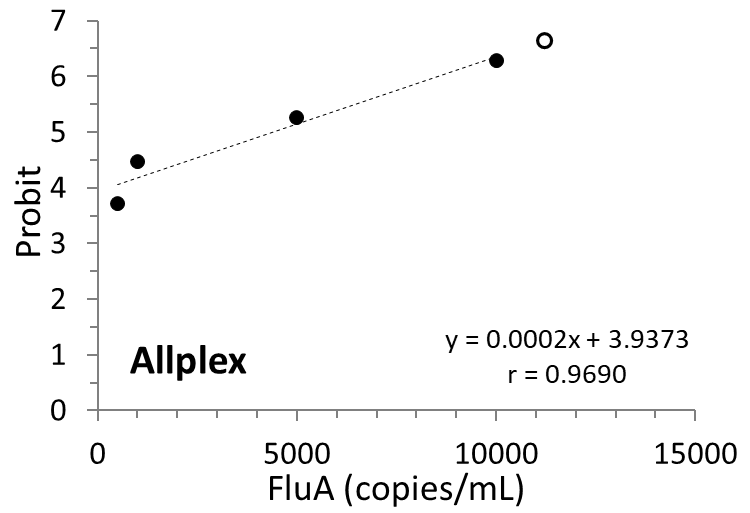
**

**C D**

**
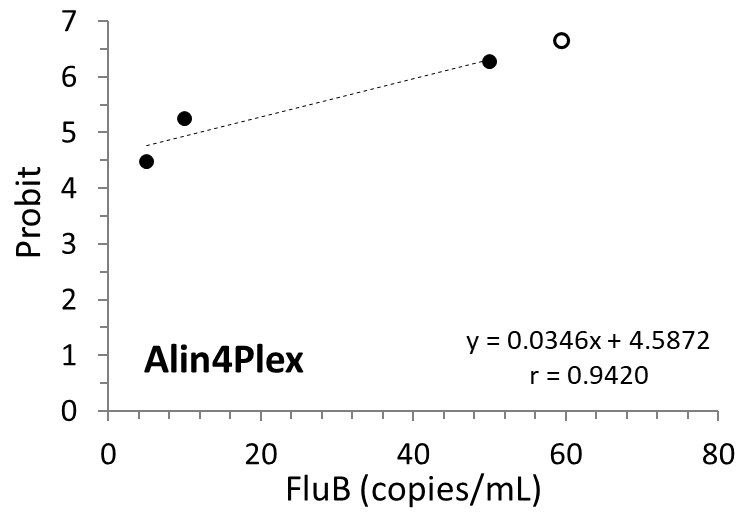

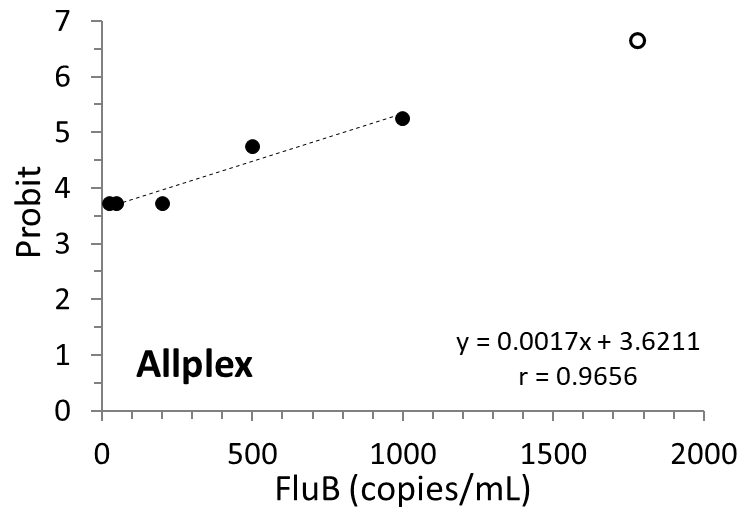
**

**E F**

**
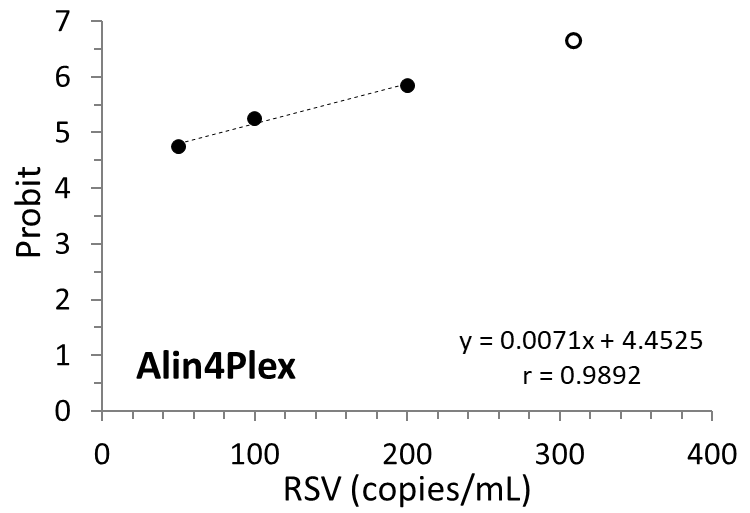

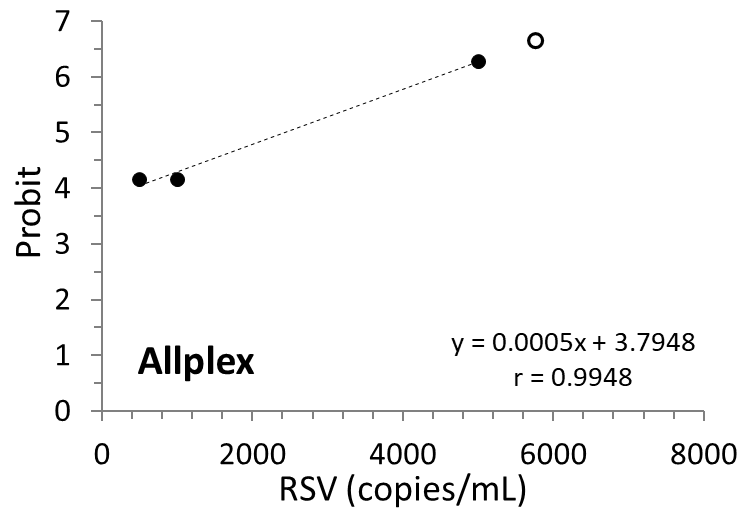
**
